# Mutation-Induced Resistance of SARS-CoV-2 M^pro^ to WU-04 Revealed by Multi-Scale Modeling

**DOI:** 10.3390/ijms27021000

**Published:** 2026-01-19

**Authors:** Mengting Liu, Derui Zhao, Hui Duan, Junyao Zhu, Liting Zheng, Nan Yuan, Yuanling Xia, Peng Sang, Liquan Yang

**Affiliations:** 1 College of Pharmacy, Dali University, Dali 671003, China; mist1368@stu.dali.edu.cn; 2 College of Agriculture and Biological Science, Dali University, Dali 671003, China; 18087176625@163.com (D.Z.); dhui4399@gmail.com (H.D.); zhujunyao@stu.dali.edu.cn (J.Z.); zlt42400326@163.com (L.Z.); yn020117@163.com (N.Y.); 3 Key Laboratory of Bioinformatics and Computational Biology, Department of Education of Yunnan Province, Dali University, Dali 671003, China; 4 Co-Innovation Center for Cangshan Mountain and Erhai Lake Integrated Protection and Green Development of Yunnan Province, Dali University, Dali 671003, China; 5 State Key Laboratory for Conservation and Utilization of Bio-Resources in Yunnan, School of Life Sciences, Yunnan University, Kunming 650091, China; xiayl@ynu.edu.cn

**Keywords:** noncovalent inhibitor, neural relational inference (NRI) model, protein-inhibitor interaction network, drug resistance mutations, WU-04

## Abstract

The clinical durability of SARS-CoV-2 main protease (M^pro^) inhibitors depends on their resilience to emerging resistance mutations. Recent genomic surveillance and functional reports have highlighted substitutions at positions 49, 165, and 301, raising questions about the robustness of the noncovalent inhibitor WU-04 in variant backgrounds. Here, we combined μs-scale, triplicate molecular dynamics simulations with end-state binding free energy estimates and a network-rewiring inference (NRI) framework that maps long-range dynamical communication across the full protease dimer. We evaluated wild type (WT), single mutants M49K, M165V, S301P, and selected double mutants (M49K & M165V, M49K & S301P). Relative to WT, single substitutions produced reductions in computed binding affinity of up to ~12kcal/mol, accompanied by loss or reshaping of the S2 subsite and altered ligand burial. Notably, the M49K/S301P double mutant partially restored WU-04 engagement, narrowing the ΔΔ*G*_restore_ gap to within ΔΔ*G*_restore_ of WT and re-establishing key hydrophobic and hydrogen-bond contacts. NRI analysis revealed that distal residue 301 participates in a communication corridor linking the C-terminal helical domain to the active-site cleft; its substitution rewires inter-domain coupling that can compensate for local disruptions at residue 49. Together, these results identify structural hotspots and network pathways that may inform the design of next-generation M^pro^ inhibitors with improved mutation tolerance—specifically by strengthening interactions that do not rely solely on the mutable S2 pocket and by engaging conserved backbone features near the 165–166 region.

## 1. Introduction

The coronavirus disease 2019 (COVID-19), caused by the severe acute respiratory syndrome coronavirus 2 (SARS-CoV-2), continues to circulate globally with ongoing case detection, hospitalizations, and deaths, and remains the subject of global public health surveillance due to persistent viral evolution and variant emergence, as documented in recent World Health Organization reports [1]. Among viral nonstructural proteins, the main protease (M^pro^, also known as 3CL^pro^) plays an essential role in viral replication by cleaving polyproteins pp1a and pp1ab at 11 conserved sites [2,3,4]. Its high sequence conservation and lack of human homologs make M^pro^ an attractive antiviral target. Several covalent and non-covalent inhibitors have been developed targeting SARS-CoV-2 M^pro^ [5,6,7,8,9,10,11], among which PF-07321332 (nirmatrelvir) has been approved for clinical use [12]. However, concerns regarding potential off-target effects and the emergence of resistance-associated mutations underscore the need for potent non-covalent inhibitors with enhanced selectivity and improved safety profiles [13,14,15]. Recently, WU-04, a novel non-covalent inhibitor with a unique chemical scaffold distinct from covalent peptidomimetics such as nirmatrelvir, has demonstrated potent preclinical activity against SARS-CoV-2 M^pro^ along with favorable pharmacokinetic properties [4,16]. Despite its promise, the structural and energetic consequences of clinically observed M^pro^ mutations on WU-04 binding remain largely unexplored.

**Figure 1:**
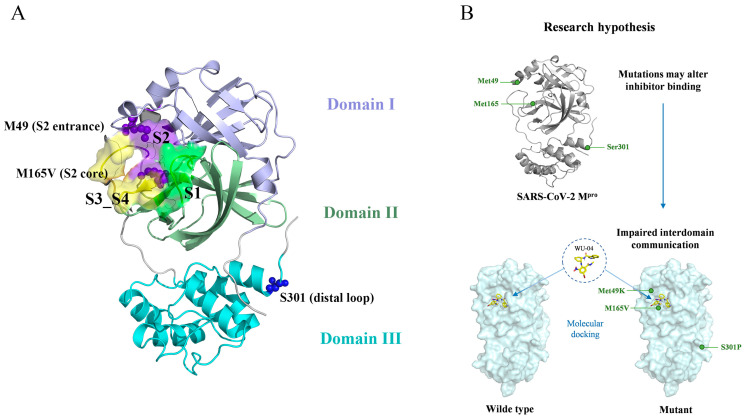
Overall structural context and research hypothesis of mutation-induced perturbations in SARS-CoV-2 Mpro. (**A**) Overall structural representation of the SARS-CoV-2 M^pro^ highlighting the S1, S2, S3_S4 region and the mutation sites analyzed in this study. The S1, S2, and S3–S4 regions are shown as semi-transparent surfaces colored green, purple, and yellow, respectively. The three structural domains are shown in distinct colors: Domain I (residues 8–101, lightpurple), Domain II (residues 102–184, pale green), and Domain III (residues 201–303, cyan). The S2–S4 region, which form a major part of the substrate- and inhibitor-binding pocket, are emphasized to provide structural context for ligand recognition. Key mutation sites are indicated, including Met49 at the entrance of the S2 pocket, Met165 at the core of the S2 region, and Ser301 located on a distal loop within Domain III. This representation provides a global spatial framework for interpreting mutation-induced alterations in local pocket dynamics and interdomain communication. (**B**) Schematic illustration of the research hypothesis. Mutations located either within the S2–S4 subsites/region (e.g., M49K and M165V) or at distal regulatory sites (e.g., S301P) may affect WU-04 binding not only through direct perturbation of local interaction networks but also by modulating long-range interdomain communication pathways. Such combined local and global effects are proposed to underlie mutation-dependent resistance and compensatory mechanisms in SARS-CoV-2 Mpro.

Although M^pro^ is more conserved than the highly mutable Spike protein and exhibits a relatively low mutation rate in circulating SARS-CoV-2 variants, large-scale genomic surveillance has revealed that key residues within or near the substrate-binding pocket (e.g., M49, M165, Q189) remain susceptible to nonsynonymous substitutions, particularly under antiviral selection pressure [17,18,19]. Despite their individually low frequencies (<0.11% across over 4.8 million genomes), these mutations raise concerns about the long-term efficacy and durability of M^pro^-targeted inhibitors [20]. Mutations within or distal to the M^pro^ active site can alter the binding affinity and resistance profile of inhibitors through direct or allosteric mechanisms [21,22]. In particular, residues such as M49, M165, and S301 have been identified as mutation hotspots associated with reduced inhibitor sensitivity [16,19,23]. Guided by the structural context of SARS-CoV-2 Mpro and the spatial distribution of mutation hotspots (Figure 1A), we hypothesize that specific point mutations within or adjacent to the M^pro^ binding pocket—such as M49K, M165V, and S301P—may perturb inhibitor binding by altering local interactions or inducing distal allosteric effects (Figure 1B). Furthermore, combinatorial mutations (e.g., M49K & M165V vs. M49K & S301P) may either exacerbate or offset resistance depending on their structural and dynamic context.

Despite extensive biochemical and crystallographic studies of M^pro^, critical knowledge gaps remain. Existing work has largely focused on covalent inhibitors and lacks a systematic evaluation of how emerging M^pro^ mutations modulate the conformational landscape, binding energetics, and allosteric communication specific to non-covalent inhibitor interactions. Moreover, current approaches rarely incorporate long-timescale dynamics or interdomain signal transduction, both of which are essential for accurately assessing mutational impacts and drug resistance potential. Without these insights, the rational design of robust, mutation-resilient inhibitors remains limited.

To fill this gap, we hypothesize that M^pro^ resistance to WU-04 arises through a multifactorial mechanism involving: (1) altered binding energetics due to pocket-lining mutations, (2) disruption of local and global conformational stability, and (3) rewiring of inter-residue and interdomain allosteric communication. To test these hypotheses, we developed a multi-scale computational framework integrating molecular docking, multi-replica microsecond-scale MD simulations, Molecular Mechanics/Poisson-Boltzmann Surface Area (MM/PBSA) binding free energy calculations, free energy landscape (FEL) profiling, residue-level signal path analysis, and graph-based network rewiring inference (NRI). This approach was applied to six clinically observed M^pro^ variants in complex with WU-04, encompassing both single and combinatorial mutations. Our results uncover mutation-specific resistance signatures and mechanistic insights into conformational destabilization, ligand reorientation, and long-range allosteric effects. These findings not only help bridge the current knowledge gap but also offer actionable guidance for the design of next-generation M^pro^ inhibitors with improved resistance profiles.

## 2. Results and Discussion

### 2.1. Molecular Docking Reveals Preliminary Binding Preferences of WU-04 Toward WT and Mutant M^pro^

Molecular docking was employed to obtain an initial view of the binding preferences of WU-04 toward SARS-CoV-2 M^pro^ WT and selected mutants. The docking scores suggested that the M49K & S301P and M49K mutants yielded slightly more favorable binding energies than the WT (Table 1), whereas the M165V displayed the weakest predicted interaction. However, these results should be interpreted cautiously. Recent biochemical reported that the M49K & S301P and M49K mutants exhibit 10–17 fold higher Kd values relative to WT [16], indicating a substantial loss of binding affinity. The apparent discrepancy likely arises from the inherent limitations of rigid docking, which neglects induced-fit effects, solvation, and long-range allosteric changes [24,25,26]. Therefore, the docking outcomes are best regarded as qualitative indicators of potential binding poses, rather than quantitative measures of affinity.

To more accurately capture the dynamic and energetic consequences of these mutations, microsecond-scale molecular dynamics simulations and MM-PBSA free-energy analyses were subsequently performed.

### 2.2. Conformational Dynamics and Interaction Persistence Under Mutation-Induced Perturbations

Analysis of backbone root-mean-square deviation (RMSD), per-residue root-mean-square fluctuation (RMSF), and hydrogen-bond persistence across three independent 1-μs trajectories revealed mutation-dependent and replica-specific effects on the conformational dynamics of the WU-04–M^pro^ complexes. To distinguish insufficient convergence from transitions among alternative metastable states, we additionally evaluated (i) per-replicate RMSD plateau behavior, (ii) overlap of conformational ensembles after 200 ns, and (iii) stability of the dominant FEL basins. All systems showed stable RMSD plateaus and substantial overlap of FEL minima across replicates, indicating that the deviations observed for certain mutants—particularly M49K and M49K & M165V—reflect transitions among neighboring metastable substates rather than incomplete trajectory convergence.

The WT complex remained compact and well-converged, with RMSD values generally ranging from 0.28 to 0.36 nm and minimal inter-replica variation (Supplementary Figure S1). In contrast, the M49K and M49K & M165V systems exhibited higher baseline RMSD values (typically 0.4–0.6 nm) and intermittent deviations reaching 0.8–1.0 nm in one trajectory, suggesting occasional transitions into alternative metastable conformations. The M49K & S301P double mutant displayed near-WT stability in two replicas, and three replicate similarly maintained a steady RMSD profile without transient excursions, indicating a consistently stabilized conformational behavior across independent simulations.

**Figure 2:**
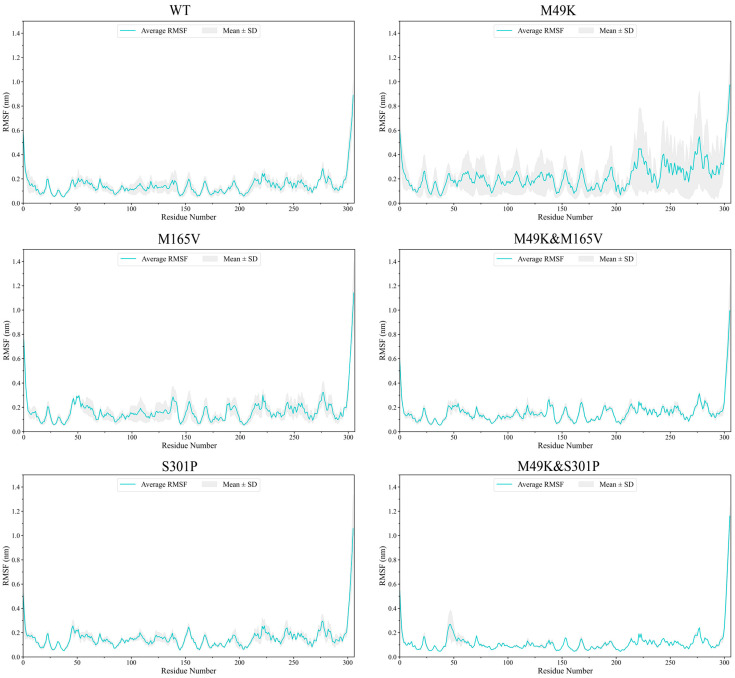
Root-mean-square fluctuation (RMSF) profiles of WU-04 bound to SARS-CoV-2 M^pro^ WT and mutant complexes during MD simulations. All MD simulations were independently repeated three times, each for 1 μs. RMSF profiles represent the average values across the three replicates, with standard deviation shown as shaded areas.

RMSF profiles (Figure 2) indicate that enhanced flexibility in the mutants is localized rather than global. Increased fluctuations in M49K and M165V primarily occur within residues 160–175 (S2 subsite) and 250–300 (inter-domain loop), two regions associated with substrate positioning and domain communication (structural overview shown in Figure 1A). These local increases in mobility were most pronounced in trajectories showing elevated RMSD.

Hydrogen-bond analysis (Supplementary Figures S2 and S3) further supports these trends. The WT complex maintained approximately three persistent ligand–protein hydrogen bonds throughout the simulations. Among all systems, M165V exhibited the lowest hydrogen-bond occupancies and lifetimes, the S301P mutant displayed the highest overall H-bond persistence, with prolonged engagement of the Glu166 and Met165 backbone atoms. The M49K and M49K & M165V mutants showed intermediate reductions in hydrogen-bond stability, whereas the M49K & S301P double mutant partially restored WT-like hydrogen-bond lifetimes, though still not fully matching the stability observed in WT or S301P.

Together, these observations correlate specific local structural perturbations with distinct dynamic responses of the WU-04–M^pro^ complexes. The M49K substitution, located at the rim of the S2 subsite, introduces both electrostatic and steric alterations that disrupt the local shape complementarity of the pocket, consistent with the increased flexibility observed in neighboring residues. The M165V mutation directly affects a core hydrophobic anchor within the S2–S4 region, leading to weakened hydrophobic packing and enhanced pocket breathing motions, as reflected by elevated RMSD and reduced hydrogen-bond persistence. In contrast, S301P resides within a distal loop connecting interdomain elements; although spatially separated from the active site, this substitution correlates with reduced interdomain mobility and reinforcement of selected backbone-mediated hydrogen bonds, thereby exerting a compensatory stabilizing influence. Collectively, these results emphasize that the observed stabilization and destabilization patterns reflect mutation-specific correlations between local structural context and global dynamics, rather than simple loss or gain of individual contacts.

### 2.3. Free Energy Landscape Profiles Highlight Destabilization and Compensatory Stabilization by Key Mutations

**Figure 3:**
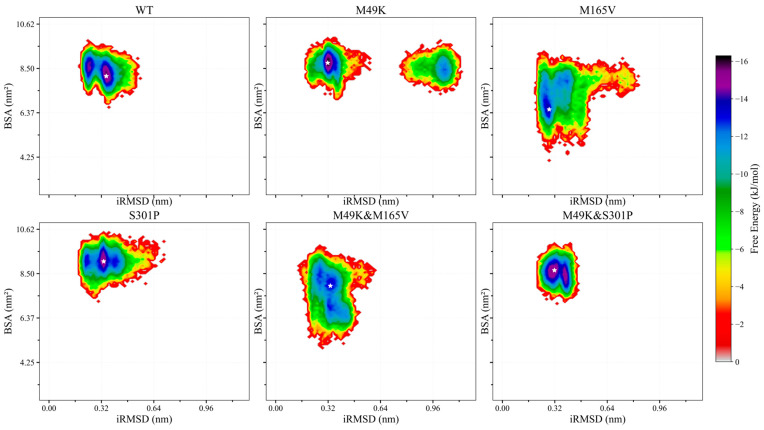
Free energy landscapes (FELs) of the six SARS-CoV-2 M^pro^–WU-04 complexes (WT, M49K, M165V, S301P, M49K & M165V, and M49K & S301P) constructed from equilibrated MD trajectories using buried surface area (BSA) and interfacial RMSD (iRMSD) as reaction coordinates. Relative free energy (kJ/mol) is indicated by the color bar.

The free energy landscapes (FELs) of the six M^pro^–WU-04 complexes were constructed using buried surface area (BSA) and interfacial RMSD (iRMSD) as collective variables to characterize the conformational stability of the binding interface (Figure 3). The WT complex displayed a single, compact low-energy basin characterized by high BSA and low iRMSD, consistent with a stable, well-packed interface. The M49K & S301P double mutant showed a similarly focused basin closely resembling that of the WT, while the S301P single mutant exhibited a slightly broadened yet still localized minimum, indicating modestly increased flexibility without loss of structural coherence.

In contrast, M165V and M49K & M165V presented markedly broadened energy surfaces with decreased BSA and increased iRMSD, reflecting impaired interface packing and reduced binding stability. The M49K mutant displayed a bifurcated FEL with two distinct low-energy basins. The primary basin closely resembled the WT conformation, whereas the secondary basin represented a metastable state with lower surface burial and greater structural deviation. This conformational heterogeneity points to a destabilizing effect of M49K on the dynamic equilibrium of the binding interface, likely driven by alterations in the S2 pocket environment.

Importantly, the M49K & S301P double mutant recovered a single, well-defined minimum on the energy surface, mirroring the WT profile and indicating structural stabilization. These findings suggest that while M49K disrupts the conformational integrity of the binding interface and introduces alternative metastable states, the S301P mutation can restore binding stability by reordering the local energy landscape. This supports a mechanism of resistance compensation driven by conformational rescue.

### 2.4. Binding Free Energy Analysis Reveals Disruption and Energetic Compensation in Mutant Complexes

To quantify the impact of key mutations on WU-04 binding to SARS-CoV-2 M^pro^, we performed MM/PBSA calculations on the WT and five mutant complexes (Table 2). The WT complex displayed the strongest binding affinity (Δ*G*_binding_ = −38.35 ± 5.43 kcal/mol), consistent with stable inhibitor engagement.

Among the single mutants, M49K and S301P led to moderate reductions in binding affinity (−35.90 ± 6.32 and −35.70 ± 7.63 kcal/mol, respectively), indicating modest disruption of intermolecular interactions. In contrast, the M165V mutant exhibited a more pronounced decrease (−23.98 ± 5.13 kcal/mol), primarily due to losses in van der Waals and electrostatic interactions, along with unfavorable solvation contributions. These findings highlight the critical role of hydrophobic packing at the S2 subsite.

Interestingly, the M49K & M165V double mutant exhibited partial energetic recovery (−26.58 ± 7.06 kcal/mol) compared to M165V alone, with improved van der Waals contacts and electrostatics, indicating limited but cooperative compensation. Most strikingly, the M49K & S301P double mutant not only retained but slightly enhanced affinity relative to WT (−38.61 ± 4.61 kcal/mol). This enhancement likely arises from a favorable balance of electrostatic and van der Waals contributions, coupled with a more moderate polar solvation penalty—implying mutual compensation between the two distal mutations.

These results underscore the differential energetic consequences of mutations and reveal that select mutation pairs, such as M49K & S301P, can restore or even improve binding through compensatory mechanisms. Such insights provide a quantitative framework for anticipating resistance risks and guiding the design of inhibitors with resilience to emerging variants.

### 2.5. RBFE Decomposition Highlights Mutation-Driven Pocket Destabilization and Compensatory Residue Interactions

**Figure 4:**
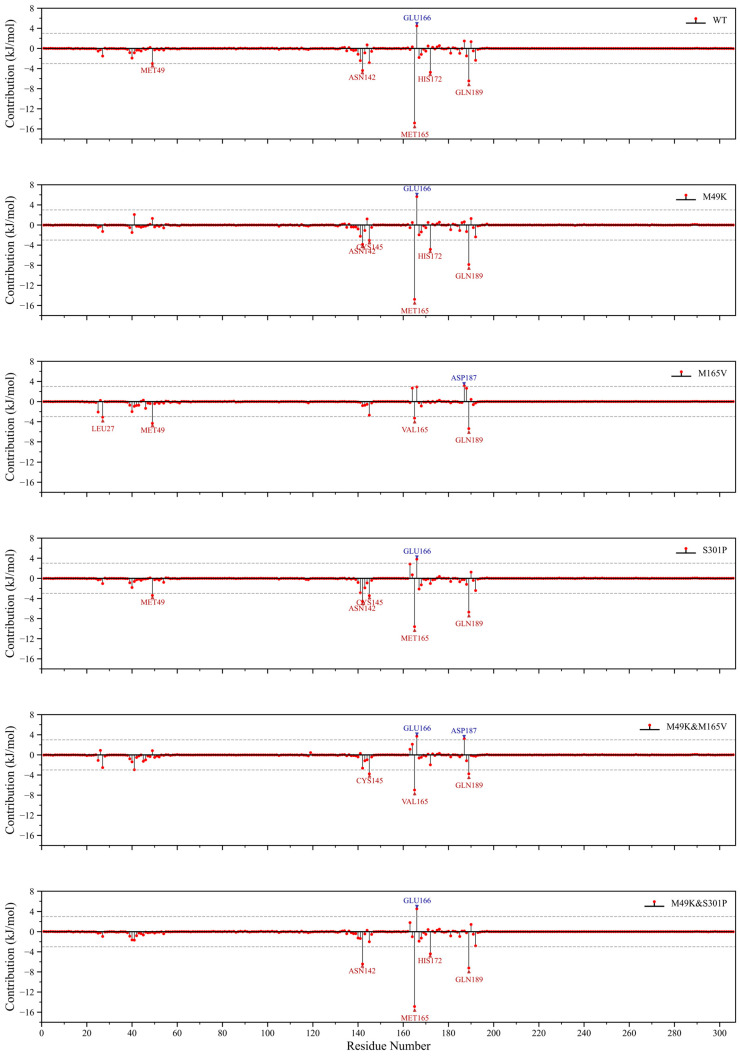
Per-residue binding free energy decomposition of WU-04 in complex with WT and mutant SARS-CoV-2 M^pro^ based on MM/PBSA calculations. MM/PBSA energy decomposition contribution between WU-04 and key residues in WT and mutant proteins. The *X*-axis is the residue number and the *Y*-axis is the free energy contribution (unit: kJ/mol). Negative values indicate favorable binding, and positive values indicate unfavorable contributions. Dashed horizontal lines mark the ±3 kJ/mol thresholds. Blue triangles highlight residues with strongly favorable contributions (ΔG < −3 kJ/mol), while red triangles indicate residues with strongly unfavorable contributions (ΔG > +3 kJ/mol).

Residue binding free energy (RBFE) decomposition was performed to pinpoint residue-specific contributions governing WU-04 recognition across WT and mutant M^pro^ complexes (Figure 4). In the WT complex, MET165, GLU166, ASN142, HIS172, and GLN189 contributed most favorably, reflecting a stable network of hydrogen-bonding and hydrophobic interactions that anchor the inhibitor within the active site. In the M49K mutant, the overall interaction pattern remained largely comparable to WT, except for a clear reduction in the local contribution from residue 49 itself, indicating that the lysine substitution selectively perturbs the immediate microenvironment of the S2 pocket without broadly altering neighboring interactions. In contrast, the M165V mutation led to a noticeable decline in stabilization from residue 165 and the emergence of an unfavorable contribution from ASP187, suggesting a subtle rearrangement of distal electrostatic contacts. The same negative contribution from ASP187 was also observed in the M49K & M165V double mutant, implying a cooperative effect that propagates destabilization toward the substrate-binding groove.

The S301P mutant preserved favorable interactions at core residues such as MET165 and GLU166 and even showed slightly enhanced contributions from ASN142 and CYS145, reflecting minimal perturbation of the binding interface. Notably, the M49K & S301P double mutant recovered favorable contributions across several key residues, yielding an energetic profile closely approaching that of WT and suggesting partial restoration of local binding stability.

Together, these results indicate that while M49K exerts a localized perturbation confined to its mutation site, M165V and M49K & M165V propagate broader destabilization through electrostatic reorganization involving ASP187. In contrast, S301P may counterbalance such destabilization by reinforcing the hydrogen-bonding and hydrophobic network around the ligand. This pattern highlights a context-dependent compensation between destabilizing and stabilizing mutations that modulates overall inhibitor affinity.

### 2.6. Representative Binding Modes Reveal Structural Basis of Mutation-Induced Alterations

**Figure 5:**
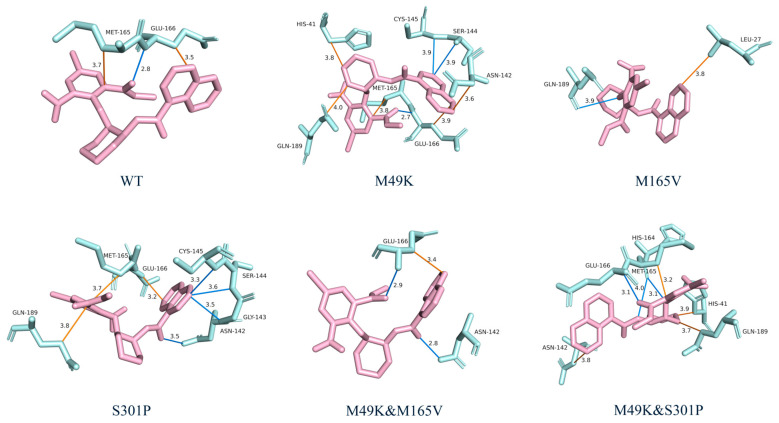
Representative binding interactions between WU-04 and SARS-CoV-2 M^pro^ in WT and mutant complexes. Key hydrogen bonds and other interactions between the inhibitor and protein residues are shown for WT, M49K, M165V, S301P, M49K & M165V, and M49K & S301P complexes. The inhibitor WU-04 is shown in pink and interacting protein residues are highlighted in light green. Distances (in Å) are labeled and interacting residues are highlighted. Hydrogen bonds are shown as marine lines, while hydrophobic interactions are shown in orange.

Representative conformations extracted from microsecond-scale MD trajectories clarified how each mutation perturbs the binding environment of WU-04 (Figure 5). In the WT complex, WU-04 maintained a compact orientation stabilized by a hydrogen bond with GLU166 (2.8 Å) and hydrophobic packing with MET165, forming a well-defined S2–S4 pocket consistent with the experimentally observed high affinity.

In the M49K mutant, the overall binding pose remained similar to WT, but local rearrangements occurred near the S2 pocket. The lysine substitution weakened the local hydrophobic packing around residue 49, consistent with the reduced RBFE contribution from M49. Interestingly, the M49K mutant exhibited a higher number of instantaneous hydrogen bonds and hydrophobic contacts with WU-04 compared to the WT. However, these additional interactions primarily arose from transient or geometrically suboptimal contacts formed upon local pocket rearrangement, rather than from stronger anchoring forces. The positively charged LYS49 side chain induced a redistribution of the S2 pocket microenvironment, allowing compensatory polar contacts but weakening the canonical backbone interactions involving MET165 and GLU166. Consequently, although the interaction counts increased, their persistence and energetic contributions were markedly reduced, consistent with the lower overall binding free energy. Hydrogen bonds with GLU166 and MET165 persisted, while transient contacts with CYS145 and ASN142 emerged. These changes indicate modest destabilization rather than complete disruption, aligning with the moderate (≈10-fold) increase in Kd [16] reported experimentally.

In contrast, the M165V substitution disrupted the key MET165 hydrophobic anchor, forcing a ligand reorientation that reduced contacts with GLU166 and GLN189. The new pose featured a weak hydrogen bond to GLN189 (3.9 Å) and peripheral hydrophobic contact with LEU27. RBFE decomposition revealed an unfavorable energetic contribution from ASP187, suggesting altered electrostatic balance in the S2–S4 region. The observed conformational dispersion is consistent with reduced binding stability and experimentally reported loss of potency. The M49K & M165V double mutant showed cumulative effects of both substitutions. Hydrogen bonds with GLU166 were weakened, the S2 pocket became more solvent-exposed, and ASP187 again displayed a negative energetic contribution. These features jointly indicate cooperative destabilization, consistent with both reduced MM-PBSA affinity and the experimentally observed lower inhibitory activity.

By contrast, the S301P mutant largely preserved the WT-like interaction network, maintaining stable hydrogen bonds with GLU166 and MET165 while introducing additional interactions with ASN142 and CYS145 that compensate for local backbone rigidity. The M49K & S301P double mutant restored a near-WT hydrogen-bonding pattern, re-establishing contacts with GLU166, MET165, and HIS164 and forming auxiliary stabilizing interactions with ASN142 and GLN189. Notably, ASP187 no longer exhibited unfavorable contributions, supporting the interpretation that S301P compensates for M49K-induced destabilization by reinforcing the inter-residue communication network.

Together, these results reconcile the computational findings with available experimental data: M49K and M165V independently weaken the binding pocket through distinct structural mechanisms, while S301P exerts a compensatory effect that partially restores interface stability. This structure–energy correspondence highlights the importance of GLU166, MET165, and GLN189 as cooperative stabilizers of the S2–S4 subsite and provides a molecular basis for understanding both resistance emergence and compensatory adaptation in SARS-CoV-2 M^pro^.

### 2.7. Allosteric Communication Network Rewiring as a Mechanism of Resistance and Compensation

**Figure 6:**
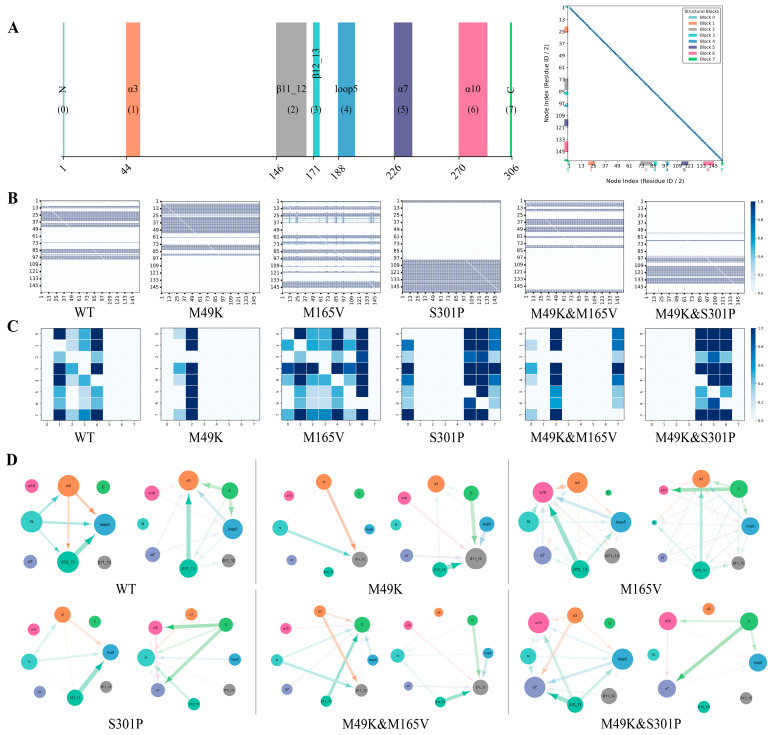
Neural Relational Inference (NRI) analysis of dynamic inter-residue and interdomain communication networks across different SARS-CoV-2 M^pro^–WU-04 complexes. (**A**) Schematic segmentation of SARS-CoV-2 M^pro^ into eight structural blocks based on secondary structure and functional regions (**left**), and mapping between the reduced residue node indices used in the NRI model and the corresponding structural blocks (**right**). During NRI model construction, residues were down-sampled by a factor of two (s = 2), while all mutation sites were explicitly retained. Color-coded bands indicate the correspondence between node indices and structural blocks and serve as reference axes for panels B–D. The diagonal line in the mapping matrix represents self-correspondence of residues or nodes within the same structural block; (**B**) Edge distributions of residue–residue interaction graphs learned from 1 μs MD trajectories using the NRI model. The diagonal elements reflect intra-block (self) interactions arising from aggregated residue–residue couplings within the same structural block, whereas off-diagonal elements indicate inter-block communication; (**C**) Aggregated domain–domain interaction matrices, showing the average learned connectivity between structural blocks 0–7 as defined in panel A. Color depth indicates interaction strength; (**D**) Directed domain-level interaction graphs constructed from the NRI-inferred dynamics. Nodes represent structural blocks, colored consistently with panel A. Directed arrows indicate the direction of inferred information flow between blocks, while edge thickness and opacity reflect the relative magnitude of the communication strength.

To probe how WU-04 binding and specific mutations reshape internal communication within SARS-CoV-2 M^pro^, we applied a Neural Relational Inference (NRI) model to 1 μs MD trajectories of six protein–ligand complexes. This model captures latent residue–residue interaction graphs over time, enabling inference of both local and global coupling pathways. To facilitate interpretation of the allosteric pathways, Figure 1A provides a global structural map of SARS-CoV-2 M^pro^, highlighting the relative spatial arrangement of functional domains, catalytic subsites (S1–S4), and the WU-04 binding pocket. All communication paths described below are therefore interpreted in the context of this structural framework. As shown in Figure 6A, the protein was partitioned into eight functionally distinct structural blocks: N-terminal (0), α3 (1), β11–12 (2), β12–13 (3), loop5 (4), α7 (5), α10 (6), and the C-terminal tail (7). These regions coordinate substrate binding, catalytic activity, and global conformational transitions.

The learned edge distributions (Figure 6B) reveal divergent communication profiles. WT retains a broadly distributed and highly interconnected interaction networks spanning loop5, α3, and the β11–12 and β12–13 strands, indicative of robust interdomain coupling. In the M165V mutant, although the global interaction network remains relatively extensive, its topology is redistributed rather than preserved, with strengthened couplings primarily observed between the β11–12 and β12–13 strands and the α10 helix, and weakened connectivity involving loop5 and α3. In contrast, M49K and S301P mutants exhibit sparse and localized interactions, implying disrupted structural coherence. Dual mutants show partially rewired yet non-redundant pathways, suggesting complex, mutation-specific remodeling. Accordingly, while both WT and M165V exhibit comparatively broader interaction networks relative to M49K, the underlying communication architectures differ, with WT maintaining loop5-centered hub organization and M165V relying on redistributed β-sheet–α10 coupling to sustain partial dynamic coherence.

Domain–domain interaction matrices (Figure 6C) further resolve these differences. In WT, loop5 acts as a central communication hub, strongly coupled to α3 and β12–13. This hub collapses in M49K, reflecting a breakdown in global coherence. M165V enhances interdomain connectivity primarily between the β11–12 and β12–13 strands and the α10 helix, while interactions involving loop5 are comparatively attenuated. This redistribution suggests that loss of local hydrophobic packing near residue 165 is partially offset by reinforcement of alternative β-sheet–helix communication routes. S301P redirects interactions toward α7 and α10, consistent with proline-induced local rigidity. While M49K & M165V shows modest recovery via re-engagement between β11–12 and loop5, the overall interaction remains directionally restricted. In contrast, M49K & S301P re-establishes a compact triad among loop5, α7, and α10, forming a spatially localized but coherent module likely stabilizing the active site. Directional communication graphs (Figure 6D) reinforce these observations. WT relies on bidirectional flux between loop5, α3, and β12–13. M49K disrupts this core axis, whereas M165V redistributes the signal landscape without fragmentation. M49K & M165V exhibits reduced redundancy, and M49K & S301P shifts the communication center to α7 and α10, forming a confined but structurally cohesive network.

Together, these results indicate that mutations can rewire allosteric communication across M^pro^, not only through local binding pocket perturbations but via global network reconfiguration. Particularly, M49K weakens substrate-domain coupling and collapses long-range interactions, potentially impairing catalysis and inhibitor engagement. Meanwhile, M49K & S301P partially restored interdomain signaling, especially between loop5, α7, and α10, supporting structural restoration and binding affinity.

These findings offer critical mechanistic insights: (1) resistance is not solely driven by direct contact loss but also by global communication breakdown; (2) effective compensatory mutations act by restoring long-range inter-block signaling, rather than merely adjusting local geometry; and (3) allosteric network remodeling, rather than static structure, better predicts mutation impact on inhibitor efficacy.

Thus, this dynamic network framework not only clarifies the mutation-induced resistance mechanisms but also provides a predictive basis for designing inhibitors resilient to allosteric disruption and variant emergence.

**Figure 7:**
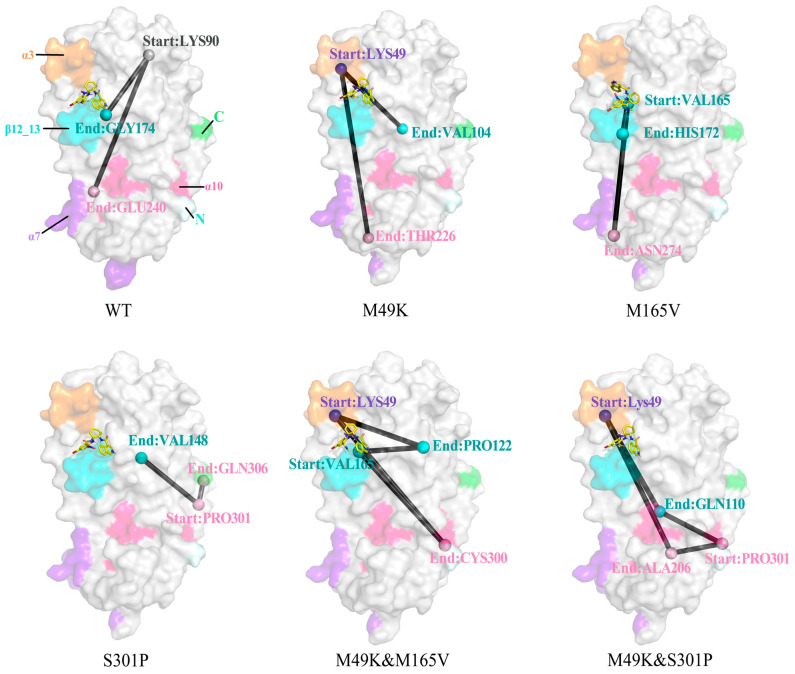
Structural projection of allosteric signal transmission pathways in WT and mutant SARS-CoV-2 M^pro^ bound to WU-04. Representative shortest allosteric communication paths are mapped onto the three-dimensional structures of WT and mutant Mpro complexes. Colored regions indicate key structural modules involved in the communication network, including the N-terminal region (light green), C-terminal region (green), α3 helix (orange), α7 helix (purple), α10 helix (hotpink), and β-strands (cyan). Dark gray lines trace the spatial routes of signal transmission, illustrating mutation-dependent rewiring of long-range allosteric pathways.

To uncover how distal mutations modulate the allosteric communication underlying WU-04 binding, we mapped residue-to-residue signal transmission pathways across WT and mutant SARS-CoV-2 M^pro^ systems (Figure 7). A comprehensive comparison of WT and mutant-specific pathways is further provided in the Supporting Information (Supplementary Figure S4), where distinct patterns of pathway rewiring, fragmentation, and redirection are observed across the five mutant backgrounds. In the WT protease, a coherent path originating from LYS90 extends through both GLY174 and GLU240, bridging β-strands and helices (α7, α10), and ensuring strong cross-domain coordination. This pathway coincides with stable inhibitor engagement and low dynamic fluctuation observed in RMSF and FEL analyses. The M49K mutant disrupts this long-range architecture. The new route initiates at LYS49, propagating only to THR226 in the α7 region. This shortened communication axis reflects local confinement of allosteric signaling and aligns with the observed loss of H-bond stability and affinity reduction, confirming M49K-induced decoupling between binding and regulatory regions. In the M165V mutant, two divergent and relatively local pathways emerge: VAL165 → HIS172 and VAL165 → ASN274. Both routes are limited to nearby structural modules and bypass interdomain helices, supporting a moderate decline in WU-04 affinity without total communication breakdown. The S301P mutant, though distal from the active site, reorganizes the allosteric flow. Two pathways originate at PRO301, reaching GLN306 and VAL148, respectively. These links suggest an alternative coupling between the C-terminal loop and protease core, explaining the mutation’s subtle yet notable effect on dynamic stabilization and binding mode reshaping. In the M49K & M165V double mutant, a combination of both mutations leads to parallel paths: LYS49 → PRO122, LYS49 → CYS300, and similarly from VAL165 → PRO122/Cys300. Despite multiple routes, all remain confined and fail to bridge to distal stabilizing elements, consistent with the system’s lowest binding free energy and pronounced conformational drift. Notably, the M49K & S301P double mutant shows the most extensive allosteric rewiring. Two sets of reciprocal paths emerge: LYS49 → GLN110 and LYS49 → ALA206, as well as PRO301 → ALA206 and PRO301 → GLN110. These converging signals reestablish spatially coherent connectivity linking the S2–S4 substrate-binding pocket (centered around residues M49, M165, and E166) with the distal α7–α10 helical bundle and the C-terminal loop, thereby reconnecting the active-site region with distal regulatory elements across domains II and III. The restored coupling aligns with improved binding stability, synergistically compensating for the M49K-induced disruption.

These allosteric pathway analyses provide a mechanistic bridge between distal mutations and observed changes in inhibitor binding affinity. While direct contact alterations explain part of the resistance, the disruption or restoration of long-range communication offers a deeper understanding of how mutations like S301P and M49K exert indirect regulatory effects. Notably, the rewired pathways in the M49K & S301P mutant highlight how compensatory dynamics can preserve inhibitor efficacy despite destabilizing single mutations. Here, “compensatory dynamics” refers to a redistribution of allosteric signal flow in which weakened local interactions caused by mutation are partially offset by strengthened coupling between alternative structural blocks, thereby preserving overall network connectivity without restoring the original WT topology. This emphasizes that drug resistance is not solely a local phenomenon but a network-level outcome, driven by both structural and dynamic reorganization. By integrating allosteric pathway tracing with binding free energy and dynamic stability assessments, our study underscores the critical need to consider global conformational communication in future antiviral design. Such insights may inform strategies to preempt resistance through targeting conserved allosteric hubs or designing inhibitors that are robust against network-level perturbations.

## 3. Materials and Methods

### 3.1. Molecular Docking of WU-04 with Wild-Type and Mutant SARS-CoV-2 M^pro^

The crystal structure of the SARS-CoV-2 M^pro^ in complex with the non-covalent inhibitor WU-04 (PDB ID: 7EN8) was used as the initial template. Six clinically observed resistance-related mutations (M49K, M165V, S301P, M49K & M165V, and M49K & S301P) [16] were introduced into the WT structure using the mutagenesis wizard in PyMOL (Schrödinger, LLC, New York, NY, USA) [27]. Molecular docking was performed using AutoDock 4.2.6 (The Scripps Research Institute, La Jolla, CA, USA) [28]. The docking grid was centered on the active site of M^pro^ with grid box dimensions of 40 Å × 40 Å × 40 Å and a grid center at coordinates (x = −26.346, y = −25.703, z = 31.226), fully encompassing the substrate-binding pocket. The Lamarckian Genetic Algorithm (LGA) [29] was employed with default settings, including Ga_runs = 50 to ensure conformational sampling. All other docking parameters were maintained at their default values.

For each complex, the top-ranked docking pose based on binding affinity was retained for further structural and energetic evaluation. This docking protocol was applied consistently across the WT and all mutant M^pro^ systems to ensure comparability.

### 3.2. Molecular Dynamics Simulations

All-atom MD simulations were carried out using GROMACS 2020.6 (GROMACS Development Team, Uppsala University, Uppsala, Sweden) [30] to investigate the conformational dynamics of WU-04 in complex with wild-type and mutant SARS-CoV-2 M^pro^. The AMBER ff99SB-ILDN force field [31] was employed for the protein, while the small-molecule inhibitor WU-04 was parameterized using AmberTools (University of California, San Francisco, CA, USA) [32]. Partial atomic charges were first assigned via the AM1-BCC [33] method using Antechamber, and the resulting topology was converted into GROMACS-compatible format using ACPYPE (Universidade de São Paulo, São Paulo, Brazil) [34]. Each system was solvated in a cubic TIP3P [35] water box with a 12 Å buffer and neutralized by Na^+^/Cl^−^ ions to 0.15 M.

300 ps of NVT equilibration at 310 K using the V-rescale thermostat [36], with heavy atoms restrained; Followed by 300 ps of NPT equilibration at 1 atm using the Parrinello–Rahman barostat [37]. Subsequently, each system underwent 1 μs of production MD, with three independent replicas per complex to ensure statistical robustness. A time step of 2 fs was used, and all covalent bonds involving hydrogen atoms were constrained via the LINCS algorithm [38]. Long-range electrostatics were treated using the Particle Mesh Ewald (PME) method with a 1.0 nm cutoff [39].

### 3.3. Stability and Key Interaction Metrics from Trajectory Analysis

To evaluate structural stability, the backbone root-mean-square deviation (RMSD) relative to the starting structure was calculated across trajectories. Local flexibility was assessed via per-residue RMSF, focusing on regions involved in ligand binding. In addition, hydrogen bonds (H-bonds) between WU-04 and M^pro^ were analyzed using the “gmx hbond” module. An H-bond was defined by a donor–acceptor distance ≤ 3.5 Å and a hydrogen–donor–acceptor angle ≥ 135°. Two metrics were computed: The number of H-bonds over time, as an indicator of dynamic contact stability; H-bond lifetime distributions, which reflect the temporal persistence of key interactions and were fitted to exponential decay models to estimate average lifetimes.

These complementary metrics (RMSD, RMSF, H-bond number, and lifetime) together provide a comprehensive assessment of the thermodynamic and structural stability of the M^pro^–WU-04 complexes under different mutation conditions.

### 3.4. Free Energy Landscapes

Two-dimensional FELs were constructed using interface RMSD (iRMSD) and buried surface area (BSA) as collective variables. The iRMSD was computed as the root-mean-square deviation of the interfacial residues (within 5 Å of the ligand) relative to the initial structure. BSA was calculated using the “gmx sasa” module by computing the solvent-accessible surface area (SASA) of the complex and its individual components (protein and ligand), and applying the relation:

(1) $$BSA=({SASA}_{protein}+{SASA}_{ligand})-{SASA}_{complex}$$

FELs were generated from concatenated trajectories (last 900 ns × 3 replicas) by binning conformations into a 2D histogram over the (iRMSD, BSA) space and converting the population distribution into free energy via the Boltzmann relation:

(2) $$G(x,y)={-k}_{B}TlnP(x,y)$$

where G(x,y) is the free energy at point (x,y), P(x,y) is the normalized probability density, $k_{B}$ is the Boltzmann constant, and T = 310 K. All FEL plots were visualized using Matplotlib v.3.10.8 (Matplotlib Development Team, USA).

### 3.5. MM/PBSA Binding Free Energy Calculations

To quantitatively evaluate the binding affinities of WU-04 to both wild-type and mutant SARS-CoV-2 M^pro^, MM/PBSA [40,41] calculations were conducted using the gmx_MMPBSA package (Valdés-Tresanco et al., Center for Molecular Simulation, Cuba) [42]. For each concatenated trajectory, a total of 270 snapshots were extracted at 10 ns intervals, ensuring adequate sampling while reducing temporal correlation between frames. The total binding free energy ($\Delta G_{binding}$) was computed using the standard MM/PBSA decomposition:

(3) $$\Delta G_{binding}=∆G_{complex}-∆G_{protein}-∆G_{ligand}$$

All calculations were carried out in implicit solvent, with a dielectric constant of 80 for the solvent and 2 for the solute [43]. The single-trajectory protocol was used to minimize conformational noise, where coordinates of the protein, ligand, and complex were extracted from the same MD trajectory. The resulting binding free energy estimates enabled a quantitative comparison of the ligand affinity across wild-type and all mutant M^pro^ complexes, providing thermodynamic support for the structural and dynamic observations.

### 3.6. Residue-Based Energy Contribution Analysis

Per-residue binding free energy decomposition was directly extracted from the MM/PBSA calculation outputs generated by the gmx_MMPBSA tool. The results include the contribution of each residue to the total binding free energy, encompassing van der Waals, electrostatic, polar solvation, and non-polar solvation terms. No additional computation was required beyond data parsing. Residues with significant energetic contributions (e.g., $\Delta G_{residue}$ ≥ ±3.0 kcal/mol) were visualized to identify key interaction hotspots and to compare energetic profiles across wild-type and mutant complexes.

### 3.7. Binding Mode Analysis of Representative Structures

For each complex, a representative frame closest to the average binding free energy was selected from the MM/PBSA ensemble. Ligand–protein interactions were visualized using Protein Ligand Interaction Profiler (PLIP) 2021 (Technische Universität Dresden, Dresden, Germany) [44] and PyMOL v.2.5.4 (Figure 5).

### 3.8. NRI for Interdomain Communication

The NRI model, a graph-based deep learning approach [45], was utilized to decode hidden inter-residue communication networks from MD simulations of wild-type and mutant SARS-CoV-2 M^pro^ complexes.

For each system, C_α_ coordinates and velocities were extracted from three 1 μs MD trajectories, subsampled to obtain 15,000 frames per system. Data were segmented into windows of 50 frames. The NRI encoder learned probabilistic interaction graphs from the trajectory data, and the decoder predicted future molecular motions based on these graphs. Model training was performed using the Adam optimizer (initial learning rate = 0.0005) over 500 epochs, and edge weights were averaged to reflect interaction probabilities. To visualize higher-level communication patterns, residues were grouped into eight structural blocks (labeled 0–7). The learned interaction matrices were coarse-grained into interdomain interaction networks, where edge width and transparency reflected the strength of dynamic communication. Weak edges (weight < 0.2) were excluded to reduce noise.

Interaction networks were visualized using Cytoscape 3.10.3 (Cytoscape Consortium, San Diego, CA, USA) [46], with edge thickness representing interaction intensity. Edges below a defined threshold (weight < 0.2) were excluded to reduce noise.

## 4. Conclusions

A detailed understanding of resistance mechanisms is crucial for developing robust, broad-spectrum antivirals against SARS-CoV-2. In this study, we constructed a multi-scale computational framework integrating molecular docking, long-timescale molecular dynamics simulations, MM/PBSA free energy calculations, residue-wise energy decomposition, free energy landscape (FEL) analysis, and signal pathway inference via network-based residue interaction (NRI) analysis. This framework enabled us to dissect how clinically relevant mutations in M^pro^ modulate its structural and energetic landscape when bound to the non-covalent inhibitor WU-04.

Our results demonstrate that mutations such as M49K and M165V modulate inhibitor binding through distinct yet converging mechanisms. M49K primarily perturbs the electrostatic and structural environment of the S2 pocket, while M165V disrupts a key hydrophobic anchor, leading to increased pocket flexibility and altered energetic contributions. These effects are most pronounced in the M49K & M165V double mutant, which exhibits marked destabilization of the active site, loss of favorable interactions involving GLU166 and MET165, and sampling of multiple metastable conformational states. In contrast, the S301P mutation, especially when combined with M49K, partially preserves native-like binding modes and dynamic stability, consistent with a compensatory effect rather than full restoration of wild-type behavior.

Beyond local binding interactions, NRI-based signal pathway analysis revealed that resistance-associated mutation induces substantial rewiring of long-range allosteric communication with M^pro^. Wild-type M^pro^ displays coherent communication pathways that couple the S2–S4 binding pocket with distal helical regions and interdomain interfaces, supporting stable ligand engagement. In resistant mutants, these pathways become fragmented or redirected, reflecting impaired cooperative dynamics and altered global regulation. This insight reveals that resistance arises not only from local perturbations at the binding interface, but also from altered intra-protein communication that compromises cooperative structural integrity.

Importantly, these allosteric network insights provide actionable guidance for the design of next-generation M^pro^ inhibitors with improved mutation tolerance. Our results suggest that robust inhibitor efficacy correlates not only with strong local binding interactions but also with preservation of long-range communication between the S2–S4 pocket and distal helical regions (α7–α10). Mutations such as M49K weaken inhibitor binding primarily by collapsing loop5-centered communication hubs, whereas compensatory substitutions (e.g., S301P) partially restore activity by reinforcing alternative interdomain pathways. These observations imply that future inhibitor design should prioritize engagement of conserved allosteric hubs (including residues such as E166 and Q189, as well as domain II–III interfaces), while avoiding over-reliance on single mutable side chains within the S2 pocket. More broadly, incorporating allosteric resilience—defined as the ability to maintain network connectivity under mutational perturbation—may represent a viable strategy to enhance inhibitor durability against emerging variants.

Altogether, our findings underscore the multifaceted nature of M^pro^ resistance, driven by both direct perturbations at the binding site and indirect conformational and allosteric rewiring. These dual mechanisms manifest through altered local interaction energetics and disrupted long-range communication networks, ultimately compromising ligand engagement and structural integrity. This highlights the critical need to incorporate protein flexibility, dynamic allostery, and interdomain coupling into the rational design of next-generation M^pro^ inhibitors. Future therapeutic strategies may benefit from stabilizing conserved subsite architectures, targeting allosteric communication hubs, or developing dual-site inhibitors that engage both orthosteric and allosteric pockets to achieve resilience against emerging resistance mutations.

## Figures and Tables

**Table 1:** Optimal docking scores of WU-04 bound to SARS-CoV-2 M^pro^ WT and resistant mutants.

Type	WT	M49K	M165V	M49K & M165V	S301P	M49K & S301P
Docking Score (kcal/mol)	−9.44	−10.75	−7.52	−8.98	−9.30	−10.79

**Table 2:** Average values and standard deviations (SDs) of the MM/PBSA energy components and binding free energies (BFE, kcal/mol) for the six SARS-CoV-2 M^pro^–WU-04 complexes (WT, M49K, M165V, S301P, M49K & M165V, and M49K & S301P), calculated from their respective MD-derived structural ensembles.

Energy Terms	Δ*E*_elec_	Δ*E*_vdW_	Δ*G*_polar_	Δ*G*_non−polar_	Δ*G*_binding_
WT	−23.88 ± 6.71	−57.94 ± 4.42	47.70 ± 5.88	−4.24 ± 0.17	−38.35 ± 5.43
M49K	−18.86 ± 6.65	−58.40 ± 4.06	45.73 ± 6.16	−4.38 ± 0.16	−35.90 ± 6.32
M165V	−10.03 ± 4.82	−41.12 ± 6.56	30.8 ± 6.48	−3.63 ± 0.40	−23.98 ± 5.13
S301P	−27.56 ± 6.64	−57.53 ± 4.39	58.53 ± 4.28	−4.57 ± 0.16	−35.70 ± 7.63
M49K & M165V	−15.85 ± 9.66	−44.33 ± 7.43	37.43 ± 10.48	−3.82 ± 0.43	−26.58 ± 7.06
M49K & S301P	−23.99 ± 6.71	−57.79 ± 3.49	51.69 ± 5.15	−4.26 ± 0.15	−38.61 ± 4.61

## Data Availability

All molecular structures, input files, and computational parameters used in this study are available in the Supporting Information (PDF). Additional data and scripts have been deposited on Zenodo (DOI: https://doi.org/10.5281/zenodo.16961795) for reviewer access.

## Supplementary Materials

The following supporting information can be downloaded at: https://www.mdpi.com/article/10.3390/ijms27021000/s1.
