# Supplementary material for: Mutation-Induced Resistance of SARS-CoV-2 Mpro to WU-04 Revealed by Multi-Scale Modeling
Source: Int J Mol Sci. 2026 Jan 19;27(2):1000. doi: 10.3390/ijms27021000 (PMC12842141; doi:10.3390/ijms27021000)
Supplement: Supplementary file 1 [file ijms-27-01000-s001.zip › ijms-3985419-supplementary.pdf]

Supporting Information for

# Mutation-Induced Resistance of SARS-CoV-2 $M^{pro}$ to WU-04 Revealed by Multi-Scale Modeling

Mengting Liu[1,2,3,4], Derui Zhao[2,3], Hui Duan[2,3], Junyao Zhu[2,3], Liting Zheng[2,3], Nan Yuan[2,3], Yuanling Xia[5], Peng Sang[2,3,4*], and Liquan Yang[2,3,4*]

[1] *College of Pharmacy, Dali University, Dali 671003, China*
[2] *College of Agriculture and Biological Science, Dali University, Dali 671003, China*
[3] *Key Laboratory of Bioinformatics and Computational Biology, Department of Education of Yunnan Province, Dali University, Dali 671003, China*
[4] *Co-Innovation Center for Cangshan Mountain and Erhai Lake Integrated Protection and Green Development of Yunnan Province, Dali University, Dali 671003, China*
[5] *State Key Laboratory for Conservation and Utilization of Bio-Resources in Yunnan and Key Laboratory of Industrial Microbial Fermentation Engineering of Yunnan Province, Yunnan University, Kunming 650091, China*

**[*] Corresponding authors**

Dr. L.Yang (ylqbioinfo@gmail.com) and Dr. P.Sang (pengsang@dali.edu.cn)

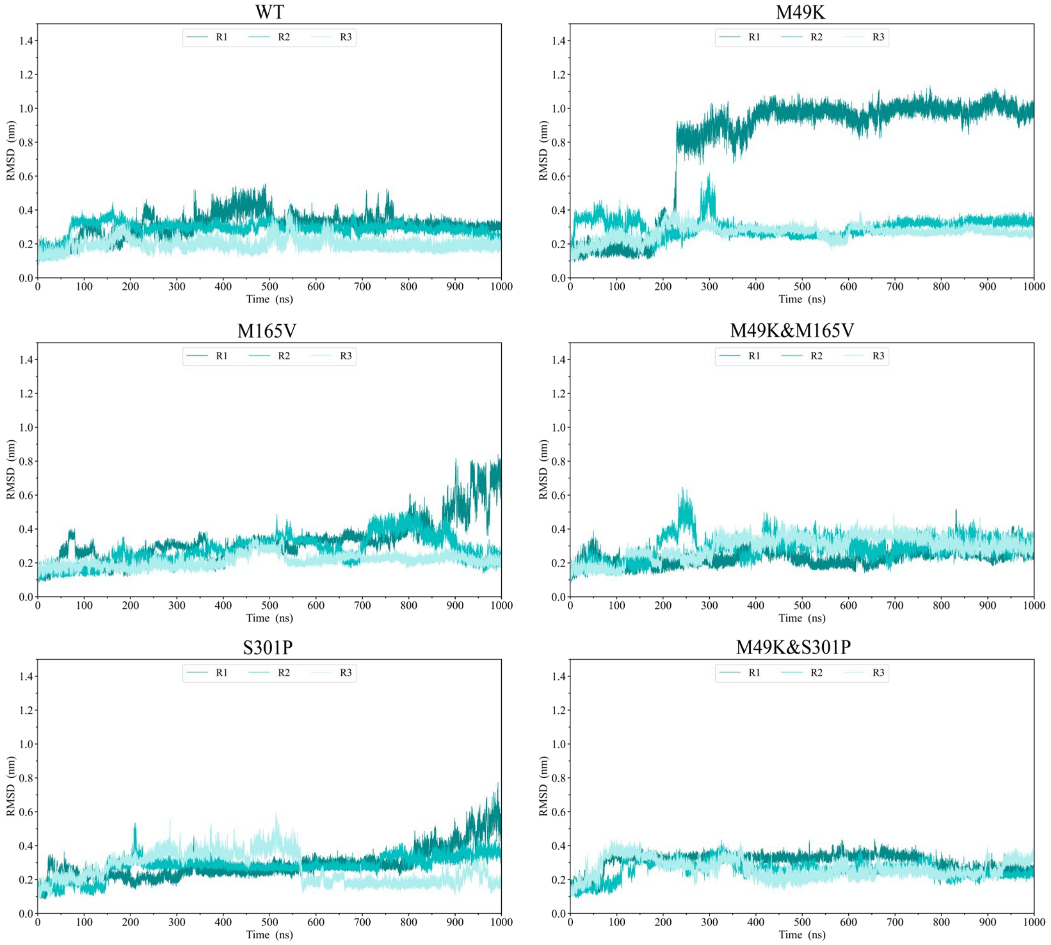


**Figure S1.** Time evolution of backbone RMSD values for wild-type and mutant SARS-CoV-2 Mpro structures during three independent 1 μs MD simulations. RMSD was computed relative to each system's initial structure, with results from replicate trajectories shown in different shades of cyan.

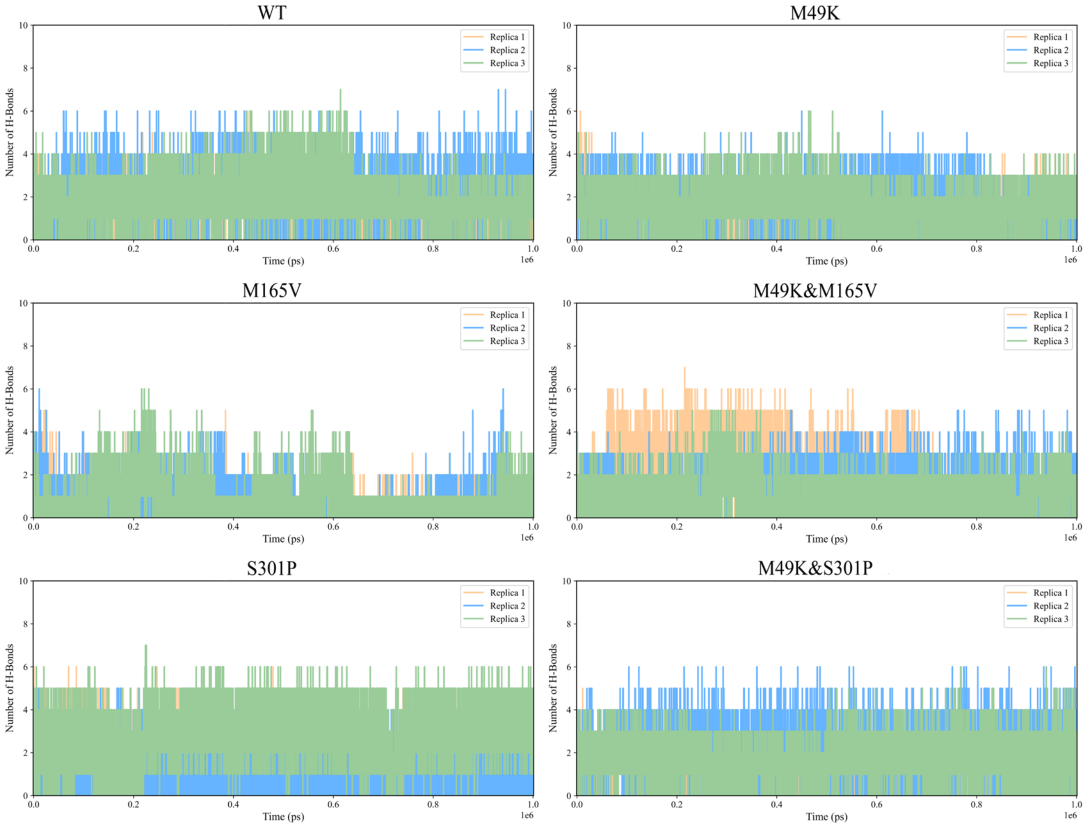


**Figure S2**. Time evolution of protein–ligand hydrogen bond numbers between WU-04 and SARS-CoV-2 Mpro. Hydrogen bond numbers were monitored across three independent 1 μs molecular dynamics simulations for WT and mutant complexes. WT and M49K&S301P maintained consistently higher H-bond counts, indicative of stable ligand engagement, whereas M49K and M49K&M165V exhibited fewer and more fluctuating H-bonds, reflecting reduced binding stability and enhanced interfacial dynamics.

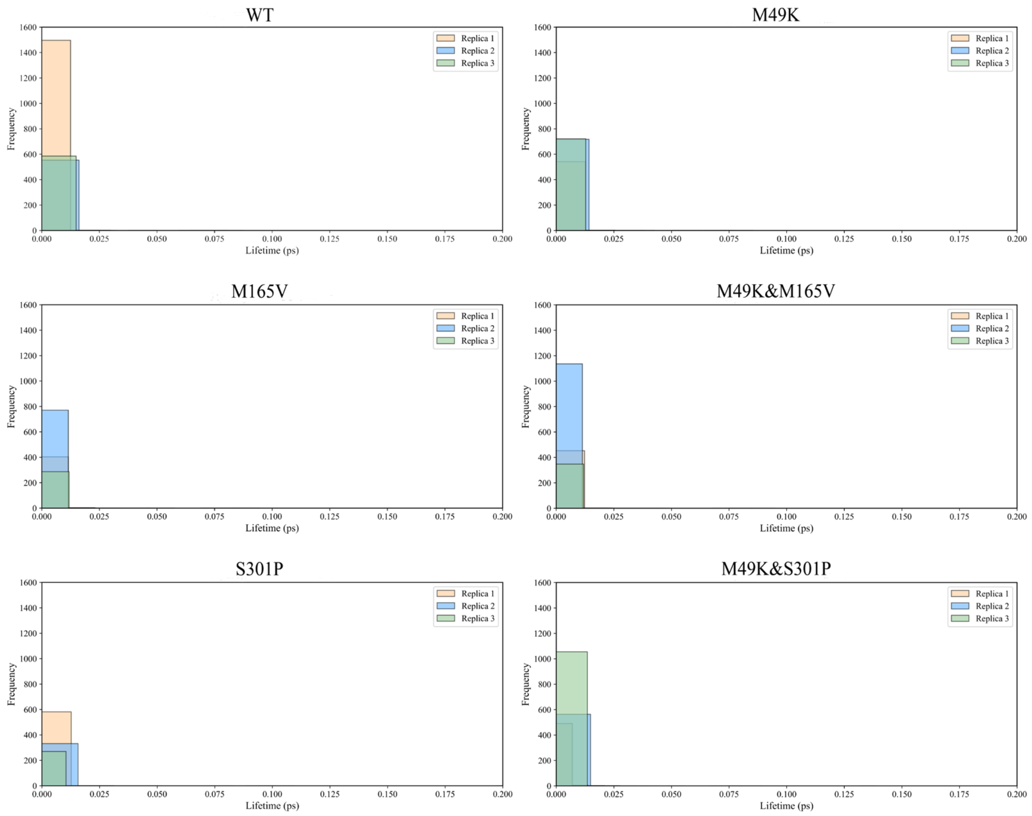


**Figure S3.** Lifetime distribution of hydrogen bonds between WU-04 and SARS-CoV-2 $M^{pro}$. Hydrogen bond lifetimes were calculated across three simulation replicas to assess the persistence of key protein–ligand interactions. WT and M49K&S301P exhibited several long-lived H-bonds, consistent with their low RMSD and stable binding conformations. M165V, M49K, and M49K&M165V showed predominantly short-lived and transient H-bonds, correlating with their reduced binding affinity and elevated conformational flexibility.

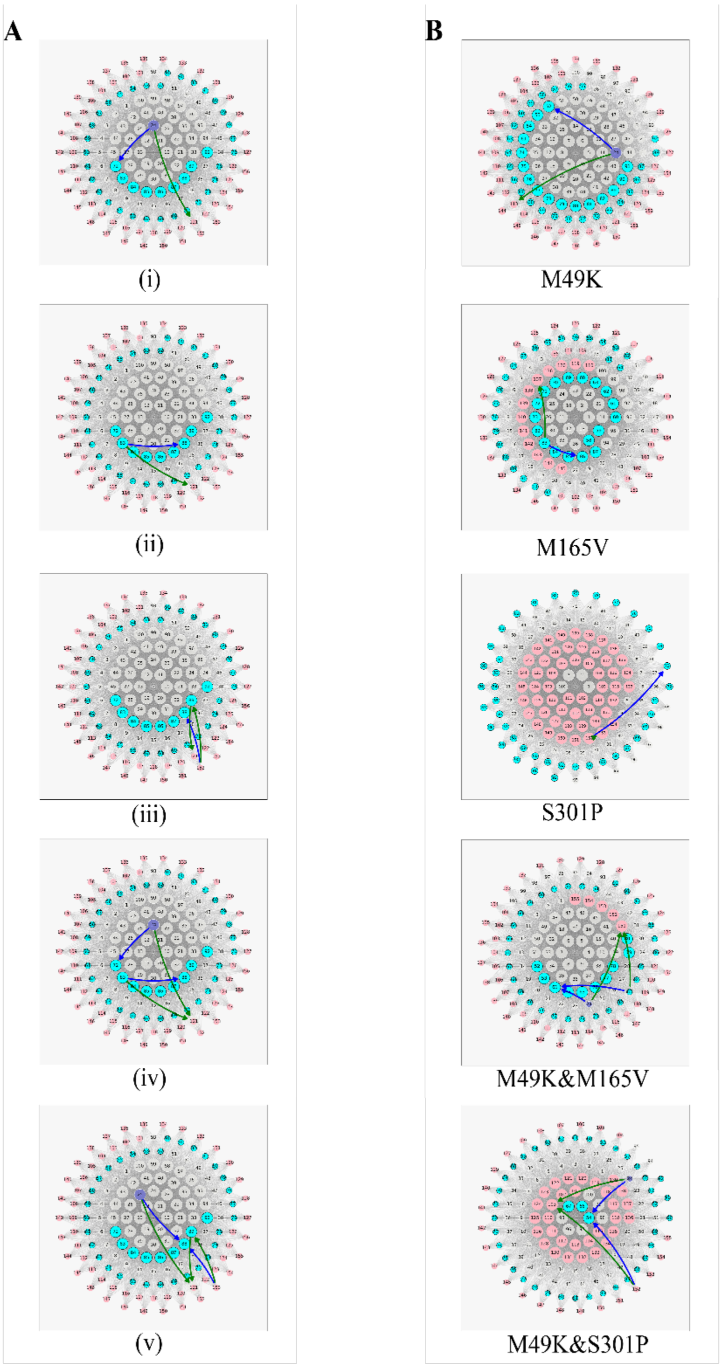


**Figure S4.** Concentric circle network diagrams of residue-level inter-domain communication in SARS-CoV-2 $M^{pro}$ variants as inferred by NRI. (A, i–v) Representative signaling pathways originating from selected seed residues in the WT SARS-CoV-2 Mpro, serving as references for mutant comparisons. (B) Signaling pathways identified in five Mpro mutants (M49K, M165V, S301P, M49K&M165V, and M49K&S301P), highlighting mutation-specific rewiring, path fragmentation, and redirection relative to the WT.

To better illustrate how mutations perturb intramolecular communication within $M^{pro}$, we constructed concentric network graphs based on the top-ranked inter-residue signaling pathways inferred by NRI (Figure S4). Panel B presents the signaling pathways identified in five individual $M^{pro}$ mutants (M49K,

M165V, S301P, M49K&M165V, and M49K&S301P), revealing varying degrees of rewiring, path fragmentation, and redirection. In contrast, Panel A (i – v) shows the corresponding pathways originating from the same seed residues in the WT background, serving as direct references for each mutant condition.

This comparative layout enables intuitive tracking of mutation-induced disruptions or compensatory adaptations in long-range communication routes. For example, M165V and M49K exhibit severely fragmented paths relative to the WT, indicative of impaired domain coupling, whereas the M49K&S301P double mutant partially restores interdomain links, suggesting a potential compensatory mechanism. These patterns collectively support the notion that resistance arises not only from direct alterations in the binding pocket but also from broader reorganization of the allosteric network architecture.
